# State‐Specific Extraction of Environmental DNA: Spike‐and‐Recovery Controls to Validate and Optimise Extraction Protocols

**DOI:** 10.1111/1462-2920.70209

**Published:** 2025-12-02

**Authors:** Julia Zöhrer, Judith Ascher‐Jenull, Eva Maria Prem, Andreas O. Wagner

**Affiliations:** ^1^ Department of Microbiology Universität Innsbruck Innsbruck Austria; ^2^ Department of Experimental Architecture, Integrative Design Extremes Universität Innsbruck Innsbruck Austria

**Keywords:** *Bacillus subtilis*, *Escherichia coli*, extracellular DNA, intracellular DNA, total environmental DNA

## Abstract

Getting insights into the quantitative and qualitative contribution of different DNA states, i.e., extracellular (exDNA) and intracellular DNA (iDNA), to the total environmental DNA (eDNA) pool requires reliable methods for their separation. Even though a multitude of respective extraction protocols has been published, their validation is often missing. Here, we selected four protocols for the state‐specific extraction of eDNA and traced the separation of exDNA and iDNA within natural environments using previously designed spike‐and‐recovery controls. Besides accounting for the different eDNA states, the spike‐ins also distinguished different bacterial origins (gram‐positive, gram‐negative). Following their quantification by digital PCR, the recovery of exDNA and iDNA spike‐ins in both the target as well as nontarget eDNA states differed among the selected extraction protocols and environmental matrices, albeit the effect of the former was far more decisive. While the recovery of exDNA spike‐ins was mainly affected by the chemical composition of the washing buffer and the duration of each washing step, the lysis method determined the recovery of spiked iDNA. These aspects were further combined within an optimised protocol, providing a valuable step towards a more concise understanding of factors governing the state‐specific extraction of eDNA and hence their relevance in molecular microbial ecology.

## Graphical abstract

Spike‐and‐recovery controls accounting for different DNA states and bacterial origins were used to validate and optimise protocols for the state‐specific extraction of environmental DNA from compost, sediment, sludge and soil samples. Despite environment‐specific differences, the recovery of spike‐ins was mainly determined by the selected extraction protocols. Hence, methodological variations related to the separation of extracellular and intracellular DNA can severely distort the outcome of eDNA‐based studies.

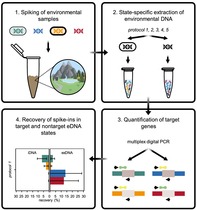

## 1 Introduction

The direct extraction and analysis of the total environmental DNA (eDNA) enables the study of microbial communities at a large scale and high taxonomic resolution. However, the total eDNA pool is made up of both intracellular DNA (iDNA) and extracellular DNA (exDNA). As the name implies, iDNA is present within structurally intact and therefore potentially living cells, while exDNA is either actively or passively released from these organisms and hence present in the extracellular environment (Levy‐Booth et al. 2007; Nagler et al. 2020; Nagler, Insam, et al. 2018; Pietramellara et al. 2009; Torti et al. 2015). In this context, it has long been assumed that the quantitative and qualitative contribution of exDNA to the total eDNA pool is negligible due to the rapid degradation of DNA molecules in the extracellular environment (Ceccherini et al. 2009; Nagler et al. 2022; Probst et al. 2021). However, growing evidence shows that exDNA may bind onto environmental surfaces, increasing its protection against enzymatic degradation and concomitantly permitting its persistence for relevant periods of time (Agnelli et al. 2004, 2007; Dell'Anno et al. 2002; Dell'Anno and Danovaro 2005; Levy‐Booth et al. 2007; Nagler et al. 2022; Nielsen et al. 2007; Ogram et al. 1994; Pietramellara et al. 2009; Torti et al. 2015). Depending on environmental conditions, exDNA may therefore represent a considerable fraction of the total eDNA pool, even exceeding the amount of iDNA (Agnelli et al. 2004; Alawi et al. 2014; Corinaldesi et al. 2005; Dell'Anno et al. 2002; Dell'Anno and Danovaro 2005; Lennon et al. 2018; Mao et al. 2014; Nagler, Podmirseg, et al. 2018; Pietramellara et al. 2009). Besides the quantitative contribution of exDNA to the total eDNA pool, however, its persistence may qualitatively bias microbiome studies, yielding false‐positive results when investigating the composition of currently prevalent microbial communities (Agnelli et al. 2004; Ascher et al. 2009; Carini et al. 2016; Ceccherini et al. 2009; Corinaldesi et al. 2018; Nagler, Insam, et al. 2018, 2021; Probst et al. 2021). For example, Carini et al. (2016) showed that exDNA inflated the observed prokaryotic and fungal richness in soils by up to 55% and 52% when compared to iDNA, respectively. Likewise, exDNA masked temporal variabilities and compositional dynamics of intact cells (i.e., iDNA) in the total eDNA pool during anaerobic digestion and the decomposition of deadwood (Nagler et al. 2021; Probst et al. 2021). Instead of analysing the directly extracted total eDNA with no possibility to distinguish between its different states, the separation of iDNA and exDNA may therefore circumvent exDNA‐dependent masking effects while revealing even more profound insights into the composition of currently prevalent microbial communities and/or past assemblages, respectively.

In this regard, a multitude of methodological approaches for the separation of different eDNA states has been proposed. For instance, exDNA molecules can be irreversibly degraded by nucleases or photoreactive chemicals such as propidium monoazide (PMA) or ethidium monoazide (EMA), restricting subsequent downstream analyses to the remaining iDNA (e.g., Albertsen et al. 2015; Burkert et al. 2019; Carini et al. 2016; Dell'Anno et al. 2002; Nocker et al. 2006; Wagner et al. 2008). In contrast, sequential washings with alkaline buffers are supposed to equally preserve exDNA and iDNA, allowing in‐depth analyses on both eDNA states (e.g., Alawi et al. 2014; Ascher et al. 2009; Corinaldesi et al. 2005; Lever et al. 2015; Mao et al. 2014; Nagler, Podmirseg, et al. 2018; Ogram et al. 1987). Indeed, the binding strength of exDNA onto environmental surfaces is reduced at alkaline pH as phosphate groups of the DNA molecules cannot bind directly to the negatively charged mineral/organic colloids. Instead, they rely on inorganic cations (e.g., K^+^, Na^+^, Mg^2+^, Ca^2+^, Al^3+^) bridging the negative charges (Levy‐Booth et al. 2007; Pietramellara et al. 2001, 2009; Torti et al. 2015). Moreover, phosphate ions in the washing solutions compete with those in the sugar‐phosphate backbone of the DNA, additionally decreasing its adsorption to the environmental surfaces (Lever et al. 2015; Pietramellara et al. 2001; Saeki et al. 2010). Even though the majority of available protocols for the recovery of both eDNA states are conceptually similar, distinct differences are apparent in terms of the separation strategy (i.e., direct vs. indirect extraction), the number of washing cycles or centrifugal forces needed to achieve the separation of the exDNA‐containing supernatant from the iDNA‐containing pellet. Furthermore, some protocols make use of additional chemicals such as polyvinylpolypyrrolidone (PVPP), sodium dodecyl sulphate (SDS), ethylenediaminetetraacetic acid (EDTA) or trypsin to optimise DNA yields, mainly in terms of exDNA (Alawi et al. 2014; Corinaldesi et al. 2005; Mao et al. 2014; Nagler, Podmirseg, et al. 2018).

**FIGURE 1:**
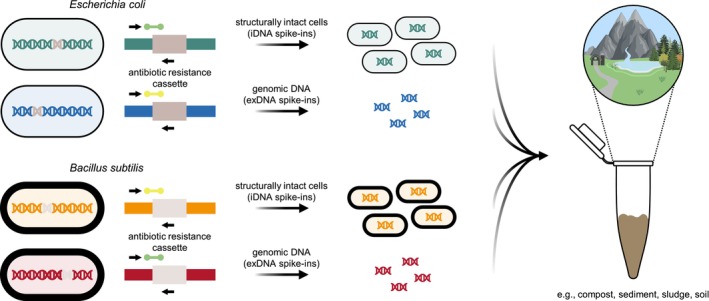
Spike‐and‐recovery controls simultaneously accounting for the different eDNA states and their bacterial origins as described previously (Zöhrer et al. 2025). Two strains of both *Escherichia coli* and *Bacillus subtilis* were selected from single‐gene deletion mutant libraries. Each of them is characterised by the deletion of a single non‐essential gene, which has been replaced by an antibiotic resistance cassette. As the genomic location of the resistance cassette is unique for each strain, targeting the terminal ends and adjacent 5′ or 3′ flanking regions allows specific quantification by PCR methods. For both bacterial organisms, one strain was used as cellular spike‐in (i.e., iDNA), while the other was subjected to genomic DNA extraction prior to its use as exDNA spike‐in. Hence, defined concentrations of all spike‐ins were simultaneously added to different environmental samples; exDNA, extracellular DNA; iDNA, intracellular DNA.

Obviously, the consequences of these methodological variations on the recovery of different eDNA states as well as the interpretation of eDNA‐based studies have hardly been assessed due to the lack of appropriate experimental setups. However, the validation and optimisation of such extraction protocols are crucial to preclude the induction of any cross‐contamination caused by cell lysis or the incomplete removal of exDNA, potentially inflating the obtained results. For instance, previous studies enumerated stained cells both prior to and after the removal of exDNA by epifluorescence microscopy or flow cytometry to monitor the occurrence of cell lysis and therefore cross‐contamination of extracted exDNA (Calderón‐Franco et al. 2021; Lever et al. 2015). Assessing the presence of insufficiently removed exDNA, however, is not feasible using this setup. Hence, the validation has mainly been confined to the use of spike‐and‐recovery controls, even though several requirements have to be met (e.g., Alawi et al. 2014; Corinaldesi et al. 2005; Kirtane et al. 2023; Mao et al. 2014; McKinney and Dungan 2020). Amongst others, the (target sequences of) spike‐ins must not occur naturally in the investigated environment and behave similarly to the focal organisms, which has proven challenging given the high microbial diversity in environmental samples. Likewise, related target genes must be easily quantified, hampering the use of genes occurring manifold within cells such as plasmids (Harrison et al. 2021; Stoeckel et al. 2009; Zemb et al. 2020). Even though several of these requirements could be circumvented by using arbitrary DNA sequences instead of living organisms (Harrison et al. 2021; Tourlousse et al. 2017; Zemb et al. 2020), appropriate spike‐and‐recovery controls for the state‐specific eDNA extraction must cover both eDNA states adequately. In this context, we previously designed spike‐and‐recovery controls simultaneously accounting for different eDNA states (i.e., exDNA and iDNA) as well as bacterial origins (i.e., gram‐negative and gram‐positive) within a single experiment. Specifically, genetically modified strains were selected from single‐gene deletion mutant libraries of two bacterial model organisms, *Escherichia coli* and *Bacillus subtilis* , being representative of a broad set of microorganisms. The genomic location of the deleted genes therefore allowed the design of specific primer/probe sets for the absolute quantification of target genes (Figure 1). Even though this methodological approach has been successfully applied to different natural environments, analyses have been restricted to the direct extraction of the total eDNA (Zöhrer et al. 2025).

Hence, the aim of the present study was to make use of these previously established spike‐and‐recovery controls to (i) validate and if necessary, (ii) optimise selected protocols for the state‐specific extraction of eDNA amongst different environments such as compost, sediment, sludge from anaerobic digestion and soil. The four selected extraction protocols represent methodological variations for the recovery of both eDNA states and have been most frequently applied in previous studies. To evaluate their performance, the percent recovery of exDNA and iDNA spike‐ins was considered in both the target eDNA state (i.e., exDNA spike‐ins recovered as exDNA, iDNA spike‐ins recovered as iDNA) as well as the nontarget eDNA state (i.e., exDNA spike‐ins recovered as iDNA and vice versa) as the latter indicates the occurrence of cross‐contamination and thus false‐positive results. Moreover, we assessed whether the state‐specific extraction can enhance the total percent recovery of spike‐ins irrespective of their presence in the target/nontarget eDNA state compared to the directly extracted total eDNA using a commercial DNA extraction kit.

## 2 Methods

### 2.1 Experimental Setup

Spike‐and‐recovery controls (Zöhrer et al. 2025) were used to validate and optimise protocols for the state‐specific extraction of eDNA within natural environments (Figure 1). Both the experimental setup as well as the selected environmental matrices (compost, sediment, sludge and soil) were previously described by Zöhrer et al. (2025). Briefly, two individual strains of both the gram‐negative *E. coli* and the gram‐positive *B. subtilis* were chosen from single‐gene deletion mutant libraries (Baba et al. 2006; Koo et al. 2017). Each of these strains is characterised by the deletion of a single non‐essential gene, which has been replaced by an antibiotic resistance cassette. Even though this resistance cassette is similar for the selected strains of each model organism, differentiation by quantitative PCR methods is possible as its genomic location is unique and thus also the terminal ends and the adjacent 5′ and 3′ flanking regions. For each model organism, one strain served as cellular spike‐in (i.e., iDNA), while the other was subjected to genomic DNA extraction prior to its use as exDNA spike‐in. This enabled us to track both eDNA states within a single experiment while concomitantly considering their bacterial origin (Figure 1, Zöhrer et al. 2025).

To ensure the application of similar and traceable amounts of iDNA and exDNA spike‐ins for all experiments, the number of cells and genome equivalents was determined within washed cell suspensions and DNA extracts, respectively. While the former was calculated by using a Thoma counting chamber (Assistant, Glaswarenfabrik Karl Hecht, GmbH & Co KG, Sondheim, Germany), the latter was determined by measuring the quantity of extracted genomic DNA (NanoDrop 2000c, Thermo Fisher Scientific, Waltham, United States), accounting also for the genomic size of the selected *E. coli* (4631 kbp) and *B. subtilis* (4215 kbp) strains. After preparing the spike‐ins, they were simultaneously added at desired concentrations to 200 mg of the selected compost, sediment, sludge and soil samples. For the state‐specific extraction of eDNA, 2.5 × 10^7^ cells and 10^7^ genome equivalents were considered appropriate for iDNA and exDNA spike‐ins, respectively. However, for the direct extraction of the total eDNA, 10^8^ cells and genome equivalents were selected for both iDNA and exDNA spike‐ins. The environmental matrices were chosen randomly, covering a variety of the most representative ecosystems for microbial community studies. Prior to the state‐specific extraction of eDNA, an adjustment period of 15 min was performed at room temperature (McKinney and Dungan 2020; Thomson‐Laing et al. 2022) to allow potential adsorption of spiked exDNA and iDNA to the environmental matrices.

### 2.2 State‐Specific Extraction of eDNA

**FIGURE 2:**
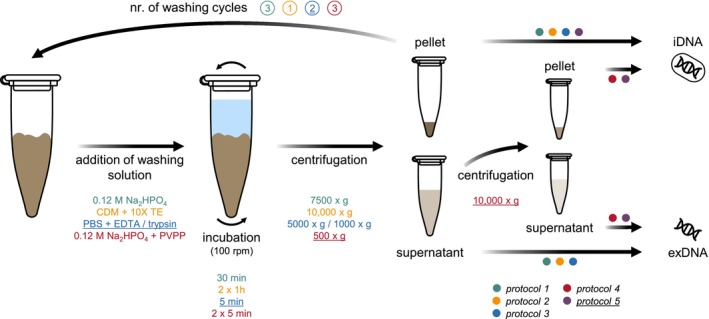
Schematic overview of the experimental workflow for the separation of exDNA and iDNA using different protocols. For all protocols, spiked environmental samples were treated with different washing solutions. After incubation, samples were centrifuged to separate the different eDNA states. For *protocols 1*, *2* and *3*, centrifugal forces ≥ 1000×*g* (i.e., high speed) were applied to separate the exDNA‐containing supernatant from the iDNA in the remaining pellet. For *protocol 4*, centrifugation at both low and high speed was required to simultaneously remove exDNA and iDNA from the environmental samples (1st centrifugation, low speed) followed by the separation of the exDNA‐containing supernatant from the remaining iDNA‐pellet (2nd centrifugation, high speed). *Protocol 5* describes a combination of *protocols 3* and *4*; therefore, the respective conditions for each step are underlined instead of listing them separately; eDNA, environmental DNA; exDNA, extracellular DNA; iDNA, intracellular DNA.

After completing an in‐depth literature review, four extraction protocols separating the different eDNA states were selected as they have been commonly applied in previous studies and concomitantly, they show apparent differences in terms of (i) the separation strategy (i.e., direct vs. indirect extraction); (ii) the composition of the washing solution; (iii) the number and duration of washing cycles; as well as (iv) the centrifugal forces applied to achieve the separation of exDNA and iDNA (Figure 2). Besides assessing the performance of these previously established extraction protocols, analyses also included a further protocol, which has been developed in this study. To ensure statistical robustness, all spike‐and‐recovery assays were performed sixfold. Consequently, simultaneous processing of all environmental samples and protocols (*n* = 120) was not feasible. Therefore, two replicates for each environmental matrix and extraction protocol (*n* = 12) were processed per day, accounting also for inter‐specific assay variation. Irrespective of the extraction protocol, all washing steps were performed within NucleoSpin Bead Tubes (Type A, Macherey‐Nagel, Düren, Germany) after removing the ceramic beads.

*Protocol 1* was conducted according to Ascher et al. (2009) with some minor modifications. Briefly, the spiked environmental samples (200 mg) were amended with 400 μL of 0.12 M Na_2_HPO_4_ (pH 8) and incubated for 30 min at room temperature on an overhead shaker (100 rpm, HulaMixer, Thermo Fisher Scientific, Waltham, United States). After centrifugation (7500×*g*, 30 min, 4°C), the exDNA‐containing supernatant was removed, while the remaining pellet was subjected to the same washing procedure for two more cycles. However, in these cases, the volume of the washing solution was reduced to 200 μL. Finally, all supernatants were pooled together and subjected to exDNA purification, while the remaining pellet was used to extract iDNA. As these processes were similar for all tested protocols, details on the purification and extraction of both exDNA and iDNA are given below.

A similar separation strategy was used within *protocol 2*, which has been previously described by Lever et al. (2015). Therefore, the spiked environmental samples (200 mg) were washed using 200 μL of a carbonate dissolution mix (0.43 M acetic acid, 0.43 M sodium acetate, pH 4.7) containing 100 mM PO_4_. After incubating the samples for 1 h on an overhead shaker (100 rpm, room temperature, HulaMixer, Thermo Fisher Scientific, Waltham, United States), 1600 μL of 10× TE buffer (pH 10) was added. Following another 1 h‐incubation period, the samples were centrifuged (10,000×*g*, 20 min, 4°C) to separate the exDNA‐containing supernatant from the iDNA‐containing pellet.

Albeit slightly modified, *protocol 3* was performed based on Nagler, Podmirseg, et al. (2018). Briefly, the exDNA was sequentially removed from the spiked environmental samples (200 mg) by applying two consecutive washing cycles. At first, the samples were amended with 1800 μL of 5 mM EDTA‐containing PBS (150 mM NaCl, 1.7 mM KH_2_PO_4_, 5 mM Na_2_HPO_4_, pH 7.4) and incubated on an overhead shaker (100 rpm, 5 min, room temperature, HulaMixer, Thermo Fisher Scientific, Waltham, United States). After centrifugation (5000×*g*, 5 min, 4°C) and the removal of the exDNA‐containing supernatant, the remaining iDNA‐containing pellet was washed using 200 μL of 0.125% trypsin‐containing PBS solution and incubated as before. Thereafter, the samples were amended with 50 μL of 0.125% trypsin‐inhibitor solution (0.125% trypsin‐inhibitor in PBS). Following another incubation period (100 rpm, 5 min, room temperature), the samples were centrifuged (1000×*g*, 5 min, 4°C) and the exDNA‐containing supernatant was removed and pooled with the previous one.

Instead of sequentially extracting exDNA and iDNA, Alawi et al. (2014) proposed a protocol simultaneously removing both eDNA states from the environmental samples prior to their separation (*protocol 4*), which is achieved by consecutive centrifugation steps at low and high speed. As suggested, the volume of the added washing solution was adjusted according to the properties of the selected environmental matrices. Hence, 800 μL 0.12 M Na_2_HPO_4_ (pH 8) and 0.05 g acid‐washed PVPP were added to the spiked environmental samples (200 mg) and incubated twice for 5 min on an overhead shaker (100 rpm, room temperature, HulaMixer, Thermo Fisher Scientific, Waltham, United States) with a chilling step on ice for 3 min in between. After centrifugation at 500×*g* (10 min, 4°C), the supernatant containing both exDNA and iDNA was removed, while the remaining pellet was resuspended in 400 μL 0.12 M Na_2_HPO_4_ (pH 8). This procedure was repeated twice and the resulting supernatants were all pooled in the same vial. After the three washing cycles, the remaining pellet was discarded, while the pooled supernatants were centrifuged at 10,000×*g* (30 min, 4°C) to separate the cells (i.e., iDNA) from the exDNA remaining in the supernatant.

*Protocol 5* was developed in this study, representing a combination of the *protocols 3* and *4* detailed above. While the composition of washing solutions, the number of washing cycles as well as their duration were chosen according to *protocol 3*, centrifugation between each washing cycle was conducted as detailed in *protocol 4*. Briefly, the spiked environmental samples were amended with 1800 μL 5 mM EDTA‐containing PBS (pH 7.4) and incubated at room temperature (100 rpm, 5 min, HulaMixer, Thermo Fisher Scientific, Waltham, United States). Next, the samples were centrifuged (500×*g*, 10 min, 4°C) and the exDNA/iDNA‐containing supernatant was removed. After adding 200 μL 0.125% trypsin solution and 50 μL 0.125% trypsin‐inhibitor solution with an incubation period (100 rpm, 5 min, room temperature) between each amendment, a further centrifugation step (500×*g*, 10 min, 4°C) was conducted. The supernatant was removed and combined with the previous one. Compared to *protocol 4*, however, the remaining pellet was not discarded. Instead, after centrifuging the pooled supernatants (10,000×*g*, 30 min, 4°C), it was combined with the resulting cell pellet to further extract iDNA, while the supernatant was subjected to exDNA purification.

#### 2.2.1 Downstream Processing of eDNA States

Even though each of the applied protocols proposed its own strategy to further process the exDNA‐containing supernatants as well as the iDNA remaining in the pellets, these steps were uniformed to enhance the comparability of the obtained results, focusing on the state‐specific extraction itself.

##### 2.2.1.1 Recovery of exDNA

The exDNA‐containing supernatants were purified using the NucleoSpin Soil Kit (Macherey‐Nagel, Düren, Germany) according to the protocol provided by the manufacturer starting at step 4 (precipitation of contaminants) to omit any steps potentially inducing cell lysis. The volumes of the buffer solutions SL3 and SB were scaled according to the volumes of the exDNA‐containing supernatants, as this was shown to improve DNA yields (*data not shown*).

##### 2.2.1.2 Recovery of iDNA

The iDNA‐containing pellets were processed according to the manufacturer's recommendations of the NucleoSpin Soil Kit (Macherey‐Nagel, Düren, Germany) with minor modifications. After adding the ceramic beads to the lysing tubes, cell disruption was performed using the FastPrep‐24 Instrument (5 m s^−1^ for 30 s, MP Biomedicals, Santa Ana, United States) in combination with Lysis Buffer SL1 and 75 μL Enhancer SX, as this was shown to achieve high recovery rates (Wagner et al. 2015).

### 2.3 Extraction of Total eDNA

The total eDNA was directly extracted from spiked environmental samples using the NucleoSpin Soil Kit (Macherey‐Nagel, Düren, Germany) as described previously (Zöhrer et al. 2025). All extractions were performed in triplicate.

### 2.4 Quantification of Target Genes

After determining the quality and quantity of the DNA extracts (i.e., exDNA, iDNA, total eDNA) by UV/VIS spectrophotometry (NanoDrop 2000c, Thermo Fisher Scientific, Waltham, United States), two multiplex digital PCR (dPCR) assays were conducted to absolutely quantify the recovered spike‐ins using the QIAcuity One, 5plex Device (Qiagen, Hilden, Germany) in combination with the QIAcuity Nanoplates 8.5 k (Qiagen, Hilden, Germany). While one assay quantified the spiked exDNA, the other specifically amplified the spiked iDNA, with each assay targeting both model organisms. The primer/probe sets, reaction setup and cycling conditions were used as described previously (Zöhrer et al. 2025). All samples were analysed at least in duplicate together with positive, negative and no‐template controls. Moreover, the percent recovery of both spiked exDNA and iDNA was calculated as shown in Zöhrer et al. (2025), considering the number of target genes recovered from the spiked environmental samples as well as the number of cells and genome equivalents used as spike‐ins.

### 2.5 Environmental Sample Properties

The content of moisture, volatile solids (VS) as well as total carbon and nitrogen was assessed as described in Zöhrer et al. (2021). Electrical conductivity (EC) and pH were determined in sample: water extracts (1:12.5, w/v) after 2 h of incubation at room temperature using a conductivity meter (LF 330 WTW, Weinheim, Germany) and a pH meter (Metrohm 826 pH mobile, Metrohm, Herisau, Switzerland), respectively. The dehydrogenase activity (DHA) as a proxy for the microbial activity was assessed by measuring the reduction of triphenyl tetrazolium chloride (TTC) to triphenylformazan (TPF) (Öhlinger and von Mersi 1996). The concentration of leachable cations (Na^+^, K^+^, NH_4_^+^, Mg^2+^) and anions (Cl^−^, NO_2_^−^, NO_3_^−^, PO_4_^3−^, SO_4_^2−^) was determined as detailed in Margreiter et al. (2024). All environmental properties were measured in triplicate.

### 2.6 Statistical Analyses

In order to evaluate the performance of the selected extraction protocols, the percent recovery of exDNA and iDNA spike‐ins in both target and nontarget eDNA states was considered. Results were therefore evaluated with regards to the eDNA states and their bacterial origins as well as the selected extraction protocols and spiked environments. Hence, non‐parametric Kruskal–Wallis tests were performed as data did not meet assumptions for parametric tests. Subsequent post hoc tests were carried out according to Dunn using Benjamini‐Hochberg‐corrected *p*‐values. In this context, the impact of environment‐ and protocol‐specific differences on the recovery of all spike‐ins was assessed using permutational multivariate analysis of variance (Adonis). Moreover, the performance of *protocol 5* was compared to its original protocols (i.e., *protocols 3* and *4*) using Wilcoxon signed‐rank tests. To further test the effect of the state‐specific extraction on total eDNA yields compared to the direct extraction using a commercial DNA extraction kit, relative changes in the percent recovery were calculated. Therefore, the percent recovery of each target gene obtained by the state‐specific extraction was subtracted from the mean percent recovery yielded by the direct extraction and divided by the latter. Regarding the analysis of physicochemical and microbiological properties, one‐way ANOVA followed by Tukey HSD tests was performed on log10(*x* + 1)‐transformed data to assess environment‐specific differences. The analyses were conducted in R v.4.4.1 (R Core Team 2024) using the packages rstatix v.0.7.2 (Kassambara 2023), reshape2 v.1.4.4 (Wickham 2007) and vegan v.2.6‐10 (Oksanen et al. 2025). Similarly, figures were created in R using the packages ggplot2 v.3.5.1 (Wickham 2016), gridExtra v.2.3 (Auguie 2017) and scales v.1.3.0 (Wickham et al. 2023). All results were considered significant at *p* ≤ 5%.

## 3 Results

### 3.1 Validation of Extraction Protocols

The performance of four different protocols for the state‐specific extraction of eDNA was evaluated within compost, sediment, sludge and soil samples using our previously designed spike‐and‐recovery controls. Overall, the percent recovery of exDNA and iDNA spike‐ins was significantly affected by both the selected extraction protocol and the environmental matrix. However, the effect of the latter was less decisive, even though the selected environments showed apparent differences regarding their physicochemical as well as microbiological properties (Table 1). For instance, considering the recovery of both exDNA and iDNA spike‐ins in the exDNA state, 38% of the explained variance was attributed to the extraction protocols (*p*_Adonis_ < 0.001), while differences between the environmental matrices accounted for 28% (*p*_Adonis_ < 0.001). The observed patterns were even more distinct for the recovery of spike‐ins in the iDNA state as the effect size related to the extraction protocols (63% of the explained variance, *p*_Adonis_ < 0.001) was more than four times higher than the effect size caused by environment‐specific differences (15% of the explained variance, *p*_Adonis_ < 0.001). Therefore, the performance of the selected extraction protocols (*protocols 1, 2, 3* and *4*) was mainly assessed independently of the environmental samples, considering both the percent recovery of spike‐ins in the target eDNA state and their cross‐contamination, i.e., detection in the nontarget eDNA state.

**TABLE 1:** Physicochemical and microbiological properties of the selected environmental matrices. Values are means (*n* = 3) with standard deviations. If applicable, they refer to the fresh weight of samples. Different letters (in brackets) indicate significant differences (*p* < 0.05) between environmental matrices.

	Compost	Sediment	Sludge	Soil
Moisture (%)	53.3 ± 0.34 (a)	24.6 ± 0.47 (b)	94.4 ± 0.13 (c)	16.3 ± 0.32 (d)
pH	8.4 ± 0.02 (a)	9.0 ± 0.05 (b)	8.2 ± 0.08 (c)	7.7 ± 0.03 (d)
EC (μS cm^−1^)	770.3 ± 2.52 (a)	49.0 ± 1.73 (b)	114.3 ± 1.53 (c)	42.3 ± 2.52 (d)
C (%)	35.1 ± 0.70 (a)	2.2 ± 0.04 (b)	27.0 ± 0.16 (c)	4.1 ± 0.26 (d)
N (%)	1.5 ± 0.02 (a)	0.0 ± 0.00 (b)	3.2 ± 0.01 (c)	0.4 ± 0.03 (d)
VS (%)	65.1 ± 0.66 (a)	0.8 ± 0.05 (b)	55.8 ± 0.16 (c)	10.7 ± 0.96 (d)
DHA (μg TFP g^−1^ 16 h^−1^)	262.0 ± 10.35 (a)	0.5 ± 0.25 (b)	77.1 ± 2.24 (c)	252.1 ± 9.76 (a)
Na^+^ (μg g^−1^)	352.9 ± 25.37 (a)	8.2 ± 1.54 (b)	103.7 ± 2.94 (c)	10.8 ± 2.01 (b)
NH_4_^+^ (μg g^−1^)	23.5 ± 13.36 (a)	0.0 ± 0.00 (b)	41.5 ± 1.23 (a)	0.0 ± 0.00 (b)
K^+^ (μg g^−1^)	2628.7 ± 125.89 (a)	42.6 ± 19.16 (b)	278.8 ± 3.54 (c)	156.1 ± 34.60 (c)
Mg^2+^ (μg g^−1^)	273.9 ± 4.49 (a)	15.1 ± 2.03 (b)	201.7 ± 11.47 (c)	140.2 ± 2.09 (d)
Cl^−^ (μg g^−1^)	1231.6 ± 12.96 (a)	656.9 ± 33.14 (b)	2029.5 ± 8.86 (c)	759.5 ± 44.72 (d)
NO_2_^−^ (μg g^−1^)	0.0 ± 0.00 (a)	862.1 ± 101.89 (b)	0.0 ± 0.00 (a)	1849.8 ± 608.00 (c)
NO_3_^−^ (μg g^−1^)	164.6 ± 24.34 (a)	180.2 ± 40.53 (a)	202.0 ± 35.00 (a)	231.5 ± 37.63 (a)
PO_4_^3−^ (μg g^−1^)	1423.6 ± 46.25 (a)	102.1 ± 9.85 (b)	2048.3 ± 799.17 (a)	178.0 ± 52.90 (b)
SO_4_^2−^ (μg g^−1^)	622.6 ± 25.33 (a)	68.1 ± 3.67 (b)	52.5 ± 4.11 (c)	53.5 ± 2.76 (c)

#### 3.1.1 Recovery of Spiked exDNA

**FIGURE 3:**
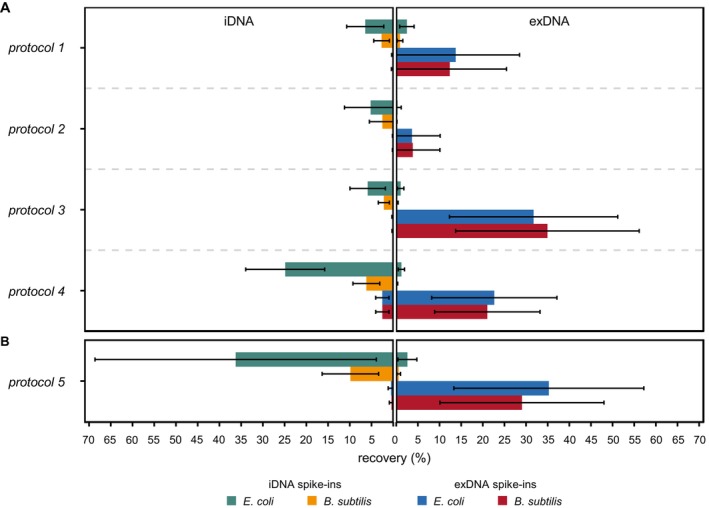
Percent recovery of exDNA and iDNA spike‐ins in both the target and nontarget eDNA state applying frequently used protocols (*1–4*) for the state‐specific extraction of eDNA (A) as well as an additional protocol (*5*) developed in this study (B); eDNA, environmental DNA; exDNA extracellular DNA; iDNA, intracellular DNA.

In terms of exDNA spike‐ins, the percent recovery of both bacterial organisms in the target eDNA state (i.e., exDNA) was highest using *protocol 3* ( *B. subtilis* : 34.9% ± 21.21%, *E. coli* : 31.7% ± 19.45%, Figure 3A). In comparison, *protocol 4* achieved similar yields ( *B. subtilis* : 21.1% ± 12.18%, *p*_Dunn_ = 0.191; *E. coli* : 22.7% ± 14.48%, *p*_Dunn_ = 0.274), while they were significantly lower for both *B. subtilis* and *E. coli* in the case of *protocol 1* ( *B. subtilis* : 12.4% ± 13.11%, *p*_Dunn_ = 0.002; *E. coli* : 13.7% ± 14.75%, *p*_Dunn_ = 0.004) and *protocol 2* ( *B. subtilis* : 3.8% ± 6.27%, *p*_Dunn_ < 0.001; *E. coli* : 3.6% ± 6.52%, *p*_Dunn_ < 0.001). Regarding their detection in the nontarget eDNA state (i.e., iDNA), three out of four tested protocols (*protocol 1, protocol 2, protocol 3*) revealed mean percent recoveries lower than 0.5% for both bacterial organisms (Figure 3A). However, for *protocol 4*, the cross‐contamination was significantly increased as 2.5% ± 1.53% and 2.5% ± 1.53% of spiked exDNA originating from *B. subtilis* and *E. coli* were erroneously extracted as iDNA, respectively. Obviously, its bacterial origin did not significantly influence the recovery of exDNA spike‐ins neither in the target nor in the nontarget eDNA state (*p*_Wilcoxon/target_ = 0.738, *p*_Wilcoxon/nontarget_ = 0.680). Accounting for differences between the environmental matrices, the performance of the selected extraction protocols changed slightly, which was most evident for sediment samples. In this case, the highest percent recovery of exDNA spike‐ins was obtained using *protocol 4* instead of *protocol 3*. However, regarding compost, sludge and soil samples, *protocol 3* was amongst those showing the highest percent recovery in the target eDNA state together with the lowest cross‐contamination (Figure S1).

#### 3.1.2 Recovery of Spiked iDNA

As opposed to exDNA spike‐ins, the percent recovery of spiked iDNA depended on its bacterial origin, revealing higher yields for *E. coli* than for *B. subtilis* in both the target (i.e., iDNA, *p*_Wilcoxon_ < 0.001) and the nontarget eDNA state (i.e., exDNA, *p*_Wilcoxon_ < 0.001). Irrespective of environment‐specific differences, *protocol 4* achieved the highest percent recovery of spiked iDNA deriving from *B. subtilis* in the target eDNA state (i.e., iDNA, 6.2% ± 3.09%), which was significantly different from *protocol 1* (2.7% ± 1.80%, *p*_Dunn_ < 0.001), *protocol 2* (2.5% ± 3.00%, *p*_Dunn_ < 0.001) and *protocol 3* (2.2% ± 1.28%, *p*_Dunn_ < 0.001, Figure 3A). Similar patterns were observed in terms of *E. coli* with *protocol 4* (24.9% ± 9.09%) showing significantly higher yields compared to the others (*protocol 1*: 6.4% ± 4.27%, *p*_Dunn_ < 0.001; *protocol 2*: 5.2% ± 6.01%, *p*_Dunn_ < 0.001; *protocol 3*: 5.9% ± 4.08%, *p*_Dunn_ < 0.001, Figure 3A). In contrast, amongst all selected extraction protocols, the mean percent recovery of iDNA spike‐ins in the nontarget eDNA state was lower or equal to 1% and 2.5% for both *B. subtilis* and *E. coli* , respectively. In the case of *protocol 2*, however, they were significantly lowest ( *B. subtilis* : 0.1% ± 0.09%, *E. coli* : 0.3% ± 0.84%, Figure 3A), albeit exceeding the percent recovery in the target eDNA state in some samples. Indeed, separate analyses for each environmental matrix changed the observed patterns slightly. Nevertheless, *protocol 4* revealed a high percent recovery of spiked iDNA in the target eDNA state together with a low cross‐contamination for compost, sludge and soil samples. For sediment, it was even the only protocol achieving a mean percent recovery > 1% for both bacterial organisms in the target eDNA state (Figure S1).

### 3.2 Optimisation of Extraction Protocols

Overall, *protocol 3* achieved the highest yields and lowest cross‐contamination in terms of exDNA spike‐ins, while *protocol 4* was well suited for the extraction of iDNA. Hence, these two extraction protocols were combined to test whether their individual benefits can be united within a single, i.e., optimised protocol (*protocol 5*). To facilitate the interpretation of the results obtained by using *protocol 5*, analyses of its performance were restricted to single comparisons with its original *protocols 3* and *4* in terms of exDNA and iDNA spike‐ins, respectively. Independent of differences between the environmental matrices, the percent recovery of spiked exDNA in the target eDNA state using *protocol 5* was similar to its original *protocol 3* for both bacterial organisms ( *B. subtilis* : 29.0% ± 18.96%, *p*_Wilcoxon_ = 0.279; *E. coli* : 35.3% ± 21.96%, *p*_Wilcoxon_ = 0.752, Figure 3B). Likewise, no significant differences were observable regarding their detection in the nontarget eDNA state ( *B. subtilis* : 0.4% ± 0.51%, *p*_Wilcoxon_ = 0.051; *E. coli* : 0.5% ± 0.66%, *p*_Wilcoxon_ = 0.087). For iDNA spike‐ins, however, *protocol 5* achieved higher yields in the target eDNA state for *B. subtilis* (9.9% ± 6.50%, *p*_Wilcoxon_ = 0.026) compared to *protocol 4*, while they were similar for *E. coli* (36.3% ± 32.37%, *p*_Wilcoxon_ = 0.214, Figure 3B). Similarly, the cross‐contamination did not differ between the combined *protocol 5* and its reference in terms of *E. coli* (2.6% ± 2.19%, *p*_Wilcoxon_ = 0.077), while it was significantly increased to 0.6% ± 0.42% for *B. subtilis* (*p*_Wilcoxon_ < 0.001, Figure 3B). Moreover, species‐specific differences in the percent recovery in both target and nontarget eDNA states were solely observed for iDNA (*p*_Wilcoxon/target_ = 0.002, *p*_Wilcoxon/nontarget_ < 0.001) but not for exDNA spike‐ins (*p*_Wilcoxon/target_ = 0.395, *p*_Wilcoxon/nontarget_ = 0.742). Evaluating the performance of *protocol 5* for each environmental matrix separately revealed slight deviations from the observed patterns, which were most evident for spiked iDNA. For instance, for compost, sludge and soil samples, significantly higher DNA yields in the target eDNA state were achieved for both *B. subtilis* and *E. coli* applying the combined *protocol 5* compared to *protocol 4*. In contrast, for sediment samples its performance was significantly reduced for both bacterial organisms as mean percent recoveries in the target eDNA state were below 1%, even exceeding the recovery in the nontarget eDNA state (Figure S1).

### 3.3 Recovery of Total eDNA

**FIGURE 4:**
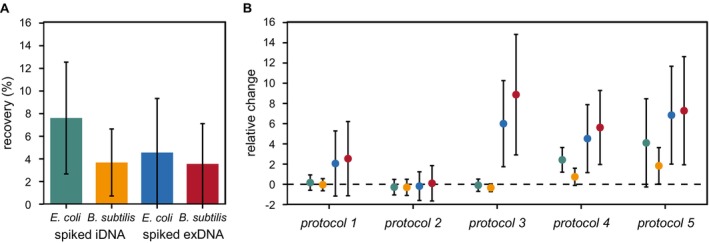
Total percent recovery of exDNA and iDNA spike‐ins by the direct extraction using a commercial DNA extraction kit (A) as well as the relative change in percent recovery obtained by each of the tested protocols for the state‐specific extraction irrespective of their detection in the target or nontarget eDNA state (B); eDNA, environmental DNA; exDNA, extracellular DNA; iDNA, intracellular DNA.

Accounting for their presence in both the target and the nontarget eDNA state, the state‐specific extraction significantly influenced the total eDNA yields compared to its direct extraction using a commercial DNA extraction kit (*p*_Kruskal‐Wallis_ < 0.001). However, the observed patterns varied between the selected protocols and spike‐ins (Figure 4). While the recovery of exDNA and iDNA spike‐ins of both *E. coli* and *B. subtilis* was similar to the direct extraction for *protocols 1* and *2*, they were significantly increased in terms of exDNA but not iDNA spike‐ins regarding *protocol 3*. In contrast, for *protocols 4* and *5*, total eDNA yields were significantly higher for both eDNA states and bacterial origins. Considering these two protocols, the increase in the total percent recovery was most apparent in terms of spiked exDNA. For instance, the recovery of exDNA spike‐ins was on average 8.3‐ and 7.8‐times higher for both *B. subtilis* (*p*_Dunn_ < 0.001) and *E. coli* (*p*_Dunn_ < 0.001) when using *protocol 5* compared to the directly extracted total eDNA (Figure 4B). Accounting also for environment‐specific differences, similar patterns were observed, albeit differently pronounced (Figure S2). Even though the recovery of iDNA spike‐ins was also significantly increased for both bacterial organisms when using *protocol 4* or *5* compared to the directly extracted total eDNA, the effect was more distinct for *E. coli* than for *B. subtilis* . For example, conducting the state‐specific extraction according to *protocol 5* increased the DNA yields of iDNA spike‐ins deriving from *E. coli* to 38.8% ± 33.18% independently of the environmental matrices, which was on average 5.1‐times higher (*p*_Dunn_ = 0.004) than those obtained by the direct extraction (Figure 4B). Under the same conditions, the increase in percent recovery of spiked iDNA from *B. subtilis* was almost half as large (2.8‐times higher, *p*_Dunn_ = 0.026). In this context, the highest percent recovery of iDNA spike‐ins was obtained for sludge samples as 86.7% ± 18.54% and 14.5% ± 1.53% of iDNA spike‐ins originating from *E. coli* and *B. subtilis* were recovered using *protocol 5*, which was on average 9.9‐ and 4.9‐times higher than by the direct extraction of the total eDNA, respectively (Figure S2).

## 4 Discussion

Even though the recovery of exDNA and iDNA spike‐ins differed amongst the selected protocols and target environments, the impact of species‐specific differences was similar for all of them. For instance, its bacterial origin significantly influenced the recovery of spiked iDNA achieving higher yields for *E. coli* than for *B. subtilis* in both target as well as nontarget eDNA states (Figure 3). Likewise, the direct extraction of the total eDNA revealed similar patterns (Figure 4A), reinforcing that gram‐positive bacteria are less prone to cell lysis. Indeed, compared to gram‐negative species, they possess a thicker cell wall due to multiple layers of crosslinked peptidoglycans, conferring higher rigidity (Albertsen et al. 2015; Auer and Weibel 2017; Frostegård et al. 1999; Nannipieri et al. 2003; Robe et al. 2003; Starke et al. 2019; Williamson et al. 2011). Compared to the selected bacterial species, many fungal organisms are even more resistant to cell lysis due to differences in their cell wall composition (Kollu and LaJeunesse 2021; Starke et al. 2019; Taubert et al. 2000). However, the implementation and accurate quantification of microbial eukaryotes as spike‐ins remain challenging as many of them show a multicellular/filamentous growth or a dimorphic lifecycle (Zöhrer et al. 2025). In contrast, species‐specific differences were not observed for spiked exDNA, emphasising that the structure and related dynamics, i.e., persistence and/or degradation, of DNA molecules in the extracellular environment are similar irrespective of their gram‐positive or gram‐negative origin.

### 4.1 Recovery of exDNA Spike‐Ins

Irrespective of the spiked environmental matrices, *protocol 3* was well suited to extract exDNA as both the recovery of exDNA spike‐ins in the target eDNA state (i.e., exDNA) was high and the concomitant cross‐contamination of the nontarget eDNA state (i.e., iDNA) was low (Figure 3A). Originally, this extraction protocol was developed for the detection of exDNA in human blood, indicating the presence of breast tumours (Laktionov et al. 2004). However, it was modified by Nagler, Podmirseg, et al. (2018) to fit its applicability to anaerobic digester sludge as well as prokaryotic organisms. Similar to the other selected extraction protocols in this study, a washing solution at both alkaline pH and high phosphate concentration was used to remove exDNA molecules bound onto organic/inorganic surfaces. In this context it was shown that the DNA yields increased with increasing pH of the washing solution (Frostegård et al. 1999; Lever et al. 2015), being even more crucial than the phosphate dose (Lever et al. 2015). However, since the protocol achieving the highest percent recovery of exDNA spike‐ins revealed the lowest pH of the washing solution, effects might be mitigated by the natural pH and the buffering capacity of the selected environmental matrices, which was above the isoelectric point of DNA molecules (pH > 5, Levy‐Booth et al. 2007) for all of them (Table 1). Under these conditions, DNA molecules possess a net negative charge, and therefore, their binding onto environmental surfaces is determined by the concentration and valence charge of cations (Levy‐Booth et al. 2007; Pietramellara et al. 2001, 2009; Romanowski et al. 1991; Torti et al. 2015). Moreover, *protocol 3* involved additional chemicals in its washing solution, i.e., EDTA and trypsin, to enhance the recovery of exDNA molecules. For instance, EDTA is a chelating agent, sequestering di‐ and trivalent metal cations such as Mg^2+^ or Ca^2+^ (Lorenz and Wackernagel 1987; Wu and Xi 2009). Despite the formation of cation‐bridges, such ions are essential co‐factors of enzymes including a broad range of nucleases (Lever et al. 2015; Pietramellara et al. 2009; Sirois and Buckley 2019). Hence, in the presence of EDTA, both the binding onto environmental particles and the degradation of exDNA molecules are inhibited, facilitating their recovery. Albeit less acknowledged, exDNA may also be associated with extracellular proteins or polysaccharides and cell surfaces (Levy‐Booth et al. 2007; Nagler, Podmirseg, et al. 2018; Pietramellara et al. 2007; Torti et al. 2015; Wu and Xi 2009). Therefore, trypsin is supposed to enzymatically release these molecules (Nagler, Podmirseg, et al. 2018). In this context, Nagler, Podmirseg, et al. (2018) distinguished three different fractions of exDNA, depending on their binding strength onto environmental particles and cell surfaces. In addition to the unbound, i.e., free exDNA, the treatments with the EDTA‐ and trypsin‐containing phosphate solutions are therefore supposed to mainly target the weakly‐ and tightly‐bound exDNA molecules, respectively. However, as the fate of DNA molecules in the extracellular environment is dynamic, the validation of this conceptual idea is hardly possible. For instance, free DNA molecules bind onto surfaces of both chemically pure minerals as well as complex environmental matrices within minutes after their addition (Blum et al. 1997; Khanna and Stotzky 1992; Lorenz and Wackernagel 1987; Romanowski et al. 1991). Despite slight deviations in the used terminology, the conceptual idea of distinguishing between free and bound exDNA molecules is also well known in aquatic environments. Therein, free exDNA is commonly referred to as dissolved DNA, while DNA bound onto environmental particles is also known as adsorbed DNA (Mauvisseau et al. 2022; Kirtane et al. 2023). Recently, Kirtane et al. (2023) developed spike‐and‐recovery controls for various water matrices simultaneously accounting for both dissolved and adsorbed DNA as well as iDNA spike‐ins representing different eukaryotic organisms. However, depending on the spiked environmental samples, the recovery of dissolved DNA spike‐ins increased when using protocols for the extraction of either adsorbed DNA or iDNA, emphasising their rapid adsorption onto organic and inorganic particles.

Besides the use of additional chemicals as essential components of the washing solution, the recovery of exDNA may also be determined by the duration of each washing step. Indeed, *protocol 3* and thus the highest recovery of exDNA spike‐ins was characterised by the shortest washing steps (i.e., 5 min) amongst all tested protocols. Prolonged incubation times may therefore increase the risk of both exDNA degradation and the co‐extraction of organic and inorganic compounds, such as humic acids, which are known to interfere with downstream analyses (Ascher et al. 2009; Daniel 2005; Nagler, Insam, et al. 2018; Nannipieri et al. 2003; Robe et al. 2003; Thomson‐Laing et al. 2022). Compared to the others, *protocol 3* also applied the lowest centrifugal forces (i.e., 1000×*g*/5000×*g*) to separate the exDNA‐containing supernatant from the iDNA‐containing pellet. However, its impact on the recovery of exDNA in both the target and the nontarget eDNA state appeared to be minor as its increase to 10,000×*g* in terms of the combined *protocol 5* did not negatively impact the obtained results (Figure 3B).

### 4.2 Recovery of iDNA Spike‐Ins

Similar to spiked exDNA, the recovery of iDNA spike‐ins of both bacterial organisms was significantly influenced by the selected extraction protocol, which was particularly evident regarding their detection in the target eDNA state (i.e., iDNA, Figure 3). Overall, the selected extraction protocols represent different strategies for the extraction of iDNA, depending on whether the structurally intact cells are directly lysed within the environmental matrices (i.e., direct extraction; *protocols 1, 2, 3*) or removed from them beforehand (i.e., indirect extraction; *protocol 4*). Indeed, these strategies are commonly known from the extraction of the total eDNA without distinguishing between its different states (e.g., Daniel 2005; Frostegård et al. 1999; Nagler et al. 2022; Nannipieri et al. 2003; Ogram et al. 1987; Robe et al. 2003; Steffan et al. 1988; Williamson et al. 2011). In this context, the direct lysis method is supposed to be more efficient, achieving higher DNA yields due to the chemical, enzymatic and/or mechanical treatment of the environmental samples, while DNA extracts are generally less contaminated and fragmented in terms of the cell separation method (Daniel 2005; Frostegård et al. 1999; Robe et al. 2003). However, results obtained by the state‐specific extraction did not coincide with these general assumptions. Instead, the recovery of iDNA spike‐ins in the target eDNA state was highest using the indirect extraction method (*protocol 4*; Figure 3A). Considering environment‐specific differences, these differences were most pronounced for sediment samples (Figure S1). Even though underlying causes remain unknown, separating the structurally intact cells from the environmental matrix may therefore also reduce their physical protection and thus enhance the cell lysis efficiency. Likewise, DNA molecules liberated during cell lysis cannot bind onto environmental particles in the case of the cell separation method, potentially increasing DNA yields (Frostegård et al. 1999; Williamson et al. 2011). Nevertheless, some structurally intact cells may still remain in the environmental matrix after their separation, as conducting the state‐specific extraction according to the optimised protocol (*protocol 5*) further increased the DNA yields of spiked iDNA deriving from both bacterial organisms for all environmental matrices except sediment (Figure S1). However, the use of the cell separation method with regard to the state‐specific extraction has rarely been considered so far, which may be related to its increased laboratory efforts. Indeed, at least two centrifugation steps at low and high speed are required to first remove both structurally intact cells and DNA molecules from the environmental matrix (i.e., low speed) followed by the separation of the exDNA‐containing supernatant from the remaining cell pellet (i.e., high speed). In this context, centrifugal forces < 1000×*g* promote the sedimentation of environmental particles, while leaving bacterial cells in the supernatant (Robe et al. 2003; Stevens and Jaykus 2004). However, due to differences in their cell size, the abundance of eukaryotes is also reduced, restricting subsequent downstream analyses to the prokaryotic share of microbial communities (Daniel 2005; Frostegård et al. 1999; Robe et al. 2003; Steffan et al. 1988). Even though analyses conducted in the present study focused on bacterial spike‐ins, this aspect needs to be considered in future studies. Furthermore, increasing centrifugal forces to separate the exDNA‐containing supernatant from the remaining cell pellet concomitantly elevates the risk of centrifugal shearing leading to the disruption of structurally intact cells and hence the cross‐contamination of extracted exDNA (Nagler, Podmirseg, et al. 2018; Pembrey et al. 1999; Peterson et al. 2012). Obviously, amongst all selected protocols, the mean percent recovery of spiked iDNA in the nontarget eDNA state (i.e., exDNA) was lower or equal to 2.5% for both bacterial organisms, emphasising that centrifugal forces ranging from 1000×*g* to 10,000×*g* did not cause major deviations due to centrifugal shearing (Figure 3).

### 4.3 Total eDNA Yields

Irrespective of environment‐specific differences, the mean percent recovery of both exDNA and iDNA spike‐ins by the direct extraction using a commercial DNA extraction kit was below 10%, indicating that the vast majority of spike‐ins was either lost during DNA extraction or could not be amplified by digital PCR (Figure 4A, Zöhrer et al. 2025). In this context, several studies reported the increase in total eDNA yields by the state‐specific extraction compared to the classical approach, whereas others did not observe this promoting effect (e.g., Alawi et al. 2014; Ascher et al. 2009; Ceccherini et al. 2009; Corinaldesi et al. 2005; McKinney and Dungan 2020; Nagler, Insam, et al. 2018, 2020, 2021; Probst et al. 2021). In fact, the state‐specific extraction significantly increased the total eDNA yields in this study as well, but not for all extraction protocols (Figure 4B). Shedding light on this controversially discussed issue, the obtained results therefore highlight the impact of methodological variations in the DNA extraction methods on the outcome of eDNA‐based studies. For instance, *protocols 4* and *5* increased the recovery of both exDNA and iDNA spike‐ins regardless of their bacterial origins, even if the increment was more pronounced for spiked exDNA (Figure 4B). In this regard, Ascher et al. (2009) suggested that the washing of environmental samples with an alkaline buffer acts as a pretreatment, simultaneously decreasing the adsorption of exDNA molecules and making microbial cell aggregates more accessible to cell lysis. Additionally, the repetition of such washing steps may further increase DNA yields (Alawi et al. 2014; Nagler, Podmirseg, et al. 2018; Robe et al. 2003; Steffan et al. 1988; Wagner et al. 2015; Williamson et al. 2011). Despite sample‐specific variations, Williamson et al. (2011) reported that the second and third rounds of extraction accounted for up to 60% of the total extractable cells from different soil types.

Indeed, the total eDNA yields and the concomitant increase related to the state‐specific extraction differed also amongst the environmental matrices selected in this study (i.e., compost, sediment, sludge and soil; Figure S2). However, deciphering underlying patterns remains challenging as mutual dependencies may obscure distinct effects of physicochemical as well as microbiological properties. In this context, it was found that the degradation of exDNA molecules decelerates at low moisture contents and pH as well as at high concentrations of salts (Nielsen et al. 2007; Pietramellara et al. 2009). The pH, in turn, is amongst the driving factors determining the influence of cations on the adsorption of DNA molecules onto environmental surfaces (Levy‐Booth et al. 2007; Pietramellara et al. 2001, 2009; Saeki et al. 2010). Moreover, the impact of environmental properties on the activity of DNA‐degrading enzymes may be limited within the present study as the state‐specific extraction of exDNA and iDNA spike‐ins started 15 min after their addition to the environmental samples. Hence, these aspects deserve further attention in future studies.

## 5 Conclusion

Answering questions regarding the ecological relevance of both exDNA and iDNA and the concomitant impact on microbial community studies requires reliable methods for the state‐specific extraction of eDNA. However, methodological variations of selected extraction methods and related consequences on downstream analyses have hardly been considered so far. Therefore, to the best of our knowledge, this study is the first of its kind validating and optimising protocols for the separation of exDNA and iDNA in natural environments using our previously designed spike‐and‐recovery controls. Within a single assay, the spike‐ins did not only account for the different eDNA states, but they also derived from both gram‐positive and gram‐negative organisms, enabling the assessment of species‐specific differences. Even though analyses were restricted to four different extraction protocols and environmental matrices, the obtained results provided valuable insights into the recovery of exDNA and iDNA. Despite slight deviations between the selected environments, the recovery of exDNA spike‐ins was determined by the chemical composition of the washing solution and the duration of the individual washing steps. In this regard, the release of exDNA molecules was favoured by their brief exposure to alkaline phosphate buffers containing chelating agents (i.e., EDTA) as well as protein‐degrading enzymes (i.e., trypsin). In contrast, the recovery of iDNA spike‐ins depended mainly on whether the structurally intact cells were lysed directly within the environmental matrix or removed beforehand, as the latter significantly increased the DNA yields. Indeed, the combination of these aspects within a single, i.e., optimised extraction protocol (*protocol 5*) revealed promising results, supporting their impact on the state‐specific extraction of eDNA. However, considering additional methods and environmental samples could further extend our understanding of underlying patterns and hence allow the development of general guidelines for their application in molecular microbial ecology.

## Author Contributions

J.Z.: conceptualization, methodology, data curation, visualisation, writing – original draft, writing – review and editing, investigation, formal analysis. J.A.‐J.: conceptualization, supervision, writing – review and editing. E.M.P.: methodology, writing – review and editing. A.O.W.: conceptualization, funding acquisition, writing – review and editing, supervision, resources, project administration. All authors read and approved the final manuscript.

## Funding

This work was supported by the Austrian Science Fund, P36711.

## Conflicts of Interest

The authors declare no conflicts of interest.

## Supporting information


**FIGURE S1:** Percent recovery of exDNA and iDNA spike‐ins in both the target and nontarget eDNA state for compost, sediment, sludge and soil samples. *Protocols 1–4* represent frequently applied extraction methods for the state‐specific extraction of eDNA, while protocol 5 represents an optimised protocol developed in this study; iDNA intracellular DNA, exDNA extracellular DNA, eDNA environmental DNA.
**FIGURE S2:** Total percent recovery of exDNA and iDNA spike‐ins by the direct extraction using a commercial DNA extraction kit for compost, sediment, sludge and soil samples. In addition, the relative change in percent recovery obtained by the state‐specific extraction of eDNA using *protocols 4* and *5* is given; iDNA intracellular DNA, exDNA extracellular DNA, eDNA environmental DNA.

## Data Availability

The data that support the findings of this study are openly available in figshare at https://doi.org/10.6084/m9.figshare.29880695.
