# Supplementary material for: State‐Specific Extraction of Environmental DNA: Spike‐and‐Recovery Controls to Validate and Optimise Extraction Protocols
Source: Environ Microbiol. 2025 Dec 2;27(12):e70209. doi: 10.1111/1462-2920.70209 (PMC12672054; doi:10.1111/1462-2920.70209)
Supplement: Supplementary file 1 — FIGURE S1: Percent recovery of exDNA and iDNA spike‐ins in both the target and nontarget eDNA state for compost, sediment, sludge and soil samples. Protocols 1–4 represent frequently applied extraction methods for the state‐specific extraction of eDNA, while protocol 5 represents an optimised protocol developed in this study; iDNA intracellular DNA, exDNA extracellular DNA, eDNA environmental DNA. FIGURE S2: Total percent recovery of exDNA and iDNA spike‐ins by the direct extraction using a commercial DNA extraction kit for compost, sediment, sludge and soil samples. In addition, the relative change in percent recovery obtained by the state‐specific extraction of eDNA using protocols 4 and 5 is given; iDNA intracellular DNA, exDNA extracellular DNA, eDNA environmental DNA. [file EMI-27-e70209-s001.docx]

**State-specific extraction of environmental DNA: Spike-and-recovery controls to validate and optimise extraction protocols**

Julia Zöhrer^1^*, Judith Ascher-Jenull^2^, Eva Maria Prem^1^, Andreas O. Wagner^1^

^1^Department of Microbiology, Universität Innsbruck, Innsbruck, Austria

^2^Department of Experimental Architecture, Integrative Design Extremes, Universität Innsbruck, Innsbruck, Austria

*Corresponding author: [julia.zoehrer@uibk.ac.at](mailto:julia.zoehrer@uibk.ac.at)

**Supplementary Material**

Supplementary Figures (Figure S1, Figure S2)

**Supplementary Figures**


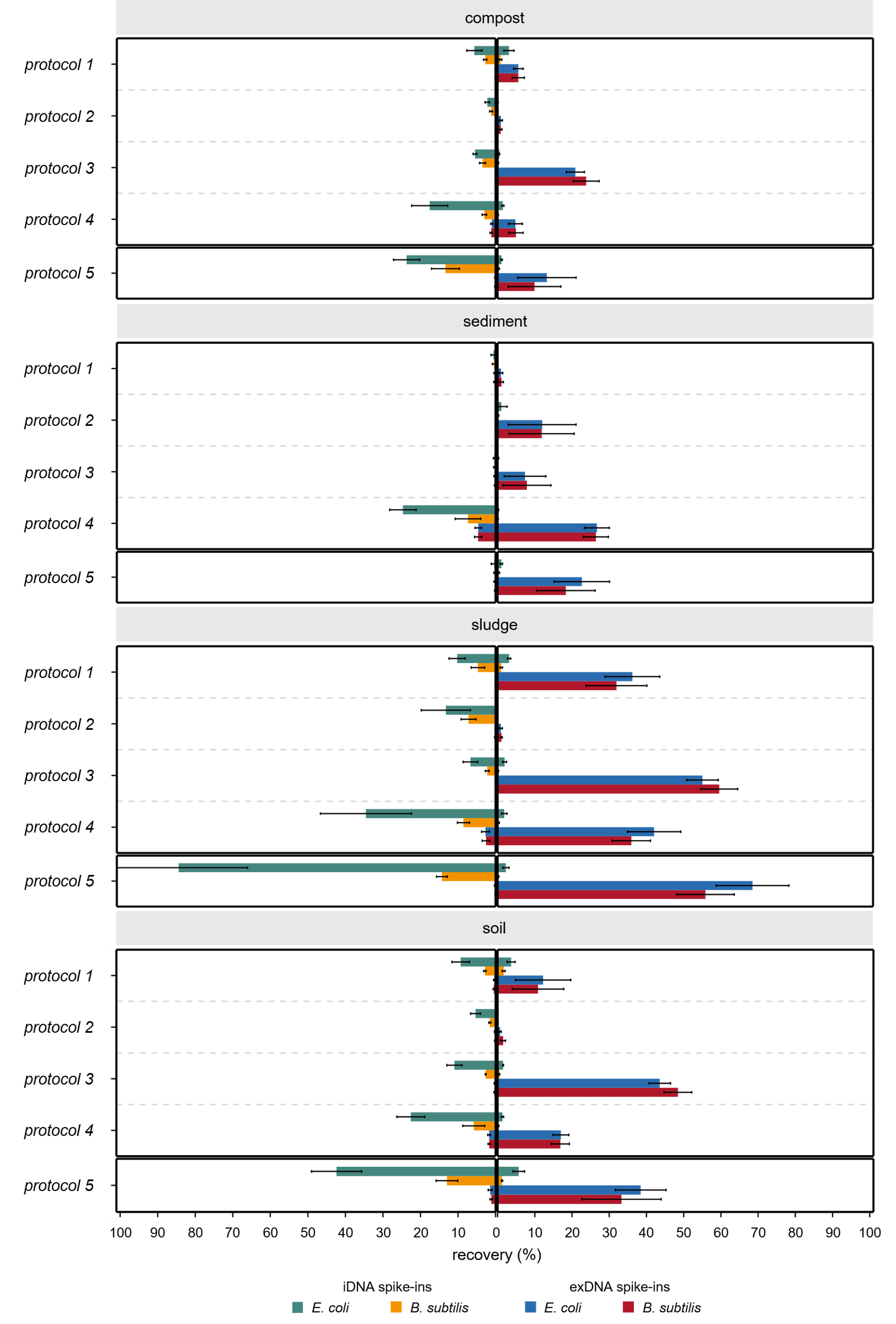


**Figure S1.** Percent recovery of exDNA and iDNA spike-ins in both the target and nontarget eDNA state for compost, sediment, sludge and soil samples. Protocols 1 – 4 represent frequently applied extraction methods for the state-specific extraction of eDNA, while protocol 5 represents an optimized protocol developed in this study; iDNA intracellular DNA, exDNA extracellular DNA, eDNA environmental DNA.


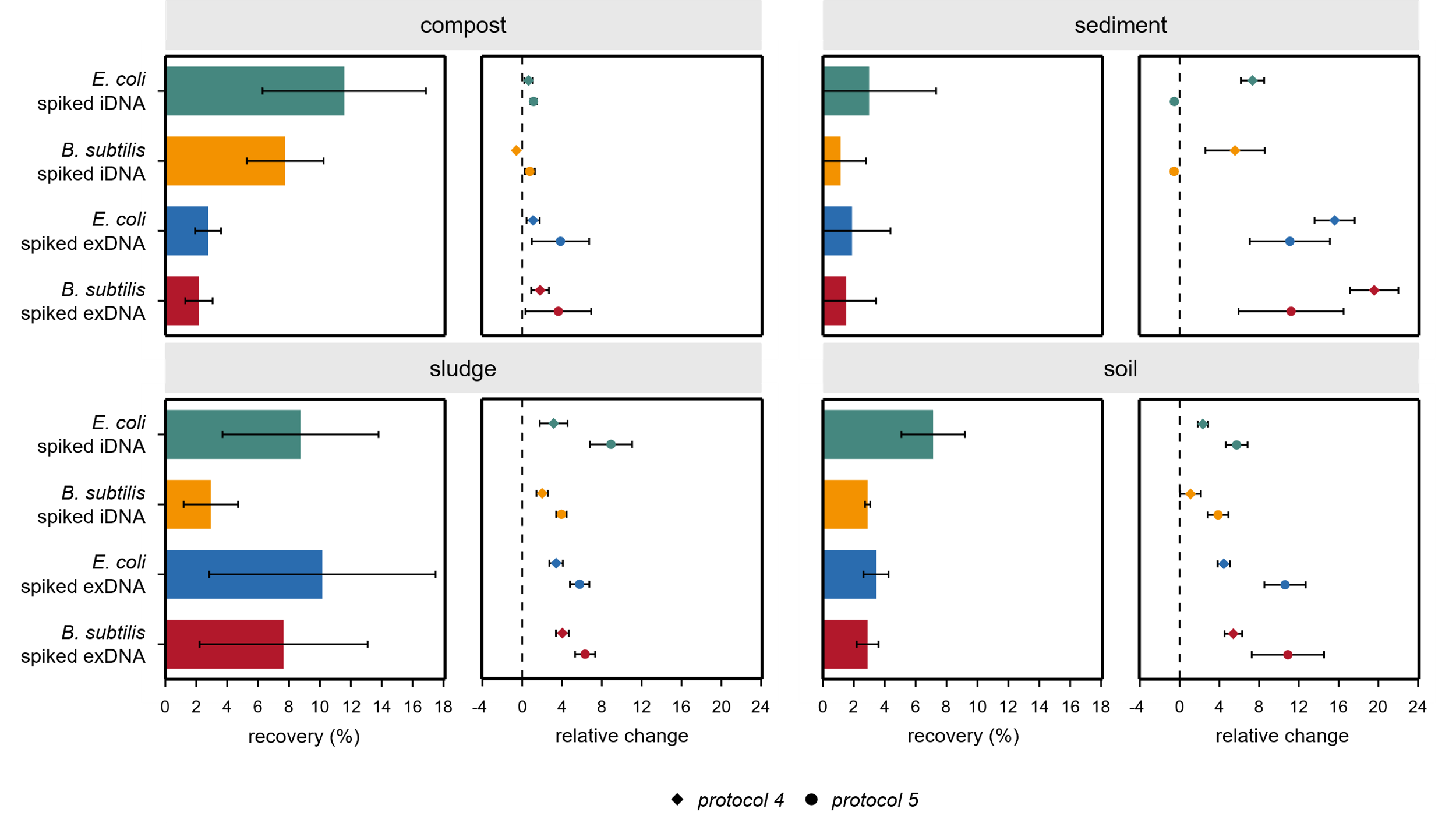


**Figure S2.** Total percent recovery of exDNA and iDNA spike-ins by the direct extraction using a commercial DNA extraction kit for compost, sediment, sludge and soil samples. In addition, the relative change in percent recovery obtained by the state-specific extraction of eDNA using protocols 4 and 5 is given; iDNA intracellular DNA, exDNA extracellular DNA, eDNA environmental DNA.
